# In vitro effect of nerve growth factor on the main traits of rabbit sperm

**DOI:** 10.1186/s12958-019-0533-4

**Published:** 2019-11-12

**Authors:** Cesare Castellini, Simona Mattioli, Alessandro Dal Bosco, Giulia Collodel, Alessandra Pistilli, Anna Maria Stabile, Lara Macchioni, Francesca Mancuso, Giovanni Luca, Mario Rende

**Affiliations:** 1 Department of Agricultural, Environmental and Food Science, University of Perugia, Borgo XX Giugno 74, 06100 Perugia, Italy; 2 Department of Molecular and Developmental Medicine, University of Siena, S. Maria dalle Scotte Hospital, 53100 Siena, Italy; 3 Section of Human, Clinical and Forensic Anatomy, Department of Surgery and Biomedical Sciences, School of Medicine, University of Perugia, P.le Lucio Severi, 1, Sant’Andrea delle Fratte, 06132 Perugia, Italy; 4 Department of Experimental Medicine, University of Perugia, P.Le Lucio Severi, 1, Sant’Andrea delle delle Fratte, 06132 Perugia, Italy

**Keywords:** NGF, Receptors, Sperm motility, Capacitation, Acrosome reaction, Apoptosis

## Abstract

**Background:**

The nerve growth factor (NGF), a member of the neurotrophins family, plays an important role not only in the nervous but also in other non-nervous systems such as the reproductive system. The aim of the paper is to study the in vitro effect of NGF on rabbit sperm functions.

**Methods:**

Ten adult rabbit bucks were collected five times, and pooled semen samples have been analysed. NGF was quantified in seminal plasma, and the distribution of NGF receptors (TrKA and p75NTR) in sperm was established. Moreover, the dose-effect of NGF on motility rate and track speed was evaluated. Successively, the effect of the neutralisation of NGF receptors was assessed to verify the specific role of each receptor. Untreated sperm were used as control.

**Results:**

Our study identified several interesting results: i) We detected NGF in seminal plasma and TrKA and p75NTR in sperm surface. In particular, TrKA is localised in the head and p75NTR in the midpiece and tail of rabbit sperm. ii) Once the optimal dose of NGF (100 ng/mL) was established, its addition affected both kinetics and other physiological traits (capacitation, apoptosis and necrosis) of rabbit sperm. (iii) The neutralisation of TrKA and p75NTR receptors affected sperm traits differently. In particular, sperm speed, apoptosis and capacitation seemed mainly modulated via p75NTR receptor, whereas motile, live cells, necrosis and acrosome reaction were modulated via TrKA.

**Conclusion:**

For the first time, we showed the presence of p75NTR in rabbit sperm. NGF affects kinetic and other physiological traits of rabbit sperm. Most of these changes are modulated by the receptors involved (TrKA or p75NTR). Considering that some seminal disorders in human have been correlated with a lower NGF concentration and no studies have been done on the possible involvement of NGF receptors, these findings also provide new insights on human fertility.

## Background

Nerve growth factor (NGF) is essential for the development, maintenance and survival of certain populations of neuronal and non-neuronal cells. The effect of NGF activity on target cells is mediated by two receptors: tropomyosin receptor kinase A (TrKA), which selectively binds NGF, and the p75 neurotrophin receptor (p75NTR), which can match up with all neurotrophin family members, including neurotrophin precursor forms. NGF binding to TrKA leads to neuronal survival, while the activation of p75NTR signalling is involved in the regulation of cell death [1–3].

NGF and its receptors TrKA and p75NTR are also widely expressed in other non-neuronal tissues like the testis, the epididymal sperm and the accessory reproductive glands [4–6]. In particular, the prostate glands of the human, guinea pig, rabbit and bull [7–12] contain a very high level of NGF. Furthermore, NGF is considered to have a functional role in sperm physiology [13], mainly affecting the fertilization process.

It is known that mature mammalian spermatozoa require capacitation in the female reproductive tract before binding to and crossing the zona pellucida and finally fusing with the oocyte plasma membrane. Defects in these processes are not detectable during sperm analysis and may represent a possible cause of idiopathic normozoospermic male infertility. At the cell biology level, capacitation induces changes in the sperm motility pattern known as hyperactivated movement and prepares the sperm to undergo an exocytotic process known as the acrosome reaction. At the molecular level, capacitation is associated with cholesterol loss from the sperm plasma membrane, increased membrane fluidity, changes in intracellular ion concentrations, hyperpolarisation of the sperm plasma membrane, increased activity of the protein kinase A (PKA) and protein tyrosine phosphorylation [14]. Defects in any of these molecular mechanisms may result in male infertility.

Several papers have shown that the NGF content in the seminal plasma of oligo- asthenozoospermic men is lower than in fertile men [15], suggesting a relevant role of NGF in sperm function.

However, the precise function and mechanism of NGF in semen remains largely undefined [9, 16], as does its role in the female reproductive tract or in sperm function [17, 18].

Recent studies have shown that the seminal plasma of many species is rich in NGF – i.e., lama and bull [13, 19], alpaca [17], and camel [20] – in both spontaneous and induced ovulatory species, because of its role in inducing ovulation [21].

Because the NGF seems to modulate several physiological traits of sperm, the aim of this paper was to verify the role of NGF and its receptors, TrKA and p75NTR, in semen traits: kinetics, capacitation, acrosome reaction and survival patterns (viable, apoptotic and necrotic) of rabbit sperm.

## Methods

If not otherwise specified, all chemicals were purchased from Sigma Aldrich (MO, USA).

### Animals and semen sampling

Ten healthy New Zealand white rabbit bucks of the same age (8 months) and weight (about 4.5 kg) were raised in the experimental farm of the Department of Agriculture, Food and Environmental Science of Perugia (Italy) and used for semen collection. Specific guidelines for rabbit bucks [22] and the International Guiding Principles for Biomedical Research Involving Animals [23] were followed. Animals were reared in compliance with the 2010/63/EU Directive transposed into the 26/2014 Legislative Decree. The experiment did not require specific authorisation by the ethical committee, because animals were not submitted to stressful treatment causing pain and suffering.

Semen collection was performed once per week by using a doelike dummy and an artificial vagina maintained at 37 °C internal temperature. Ten consecutive semen collections were conducted in April–June 2018.

#### Semen handling

Immediately after semen collection, the sperm concentration was measured using a Thoma–Zeiss cell counting chamber and a light microscope (Olympus CH_2_, Japan) set at 40X objective magnification. An aliquot of each semen sample (concentration > 350 × 10^6^ cells/mL and motility rate > 85%) for each collection was stored individually for western blotting analysis, while the rest was pooled and divided into different aliquots and diluted with a modified TALP/BSA (composed of 5.69 g/L NaCl, 0.23 g/L KCl, 0.29 g/L CaCl_2_·2H_2_O, 0.08 g/L MgCl_2_·6H_2_O, 0.04 g/L Na_2_HPO_4_, 2.09 g/L NaHCO_3_, 0.02 g/L sodium pyruvate, 0.37% lactic acid, 2.38 g/L HEPES, 50 mg/L gentamycin and 1% bovine serum albumin – BSA) to achieve a final concentration of 10^8^ sperm/mL. Its osmolarity and pH values were 296 mOsm/kg and 7.4, respectively.

### Experimental design

Three different experiments were performed to evaluate the role of NGF on different sperm traits. In particular, the following trials have been executed:

1. Quantification of NGF in seminal plasma and analysis of TrKA and p75NTR receptors in ejaculated sperm: An aliquot of semen (about 1 mL) from the pooled sample at each collection was centrifuged at 700 x g for 15 min to obtain seminal plasma (SP). TrKA and p75NTR receptors in sperm were identified with different techniques (immunolocalisation; FACSscan, western blot), illustrated later. Both receptors were analysed in raw and cells undergoing acrosome-reaction (AR). AR was induced using 5 μM (200 μL) calcium ionophore, according to Carretero et al. [24].
2. Dose-effect of NGF on ejaculated sperm: To determine the optimal dose of NGF (human NGF, from 50 to 200 ng/mL) on the in vitro motility rate and track speed of sperm, the sperm suspensions were treated with different doses of NGF and analysed after 30 min of incubation under 5% CO_2_ at 37 °C. Three successive replications were performed.
3. Neutralisation of NGF receptors (TrKA and p75NTR) and semen traits: We carried out preliminary trials to define the suitable doses of TrKA antibody (AF175 R&D Systems, MN, USA; from 8 to 50 μg/mL) and p75NTR antibody (Me20.4 Monoclonal Antibody Millipore, CA, USA; from 0.8 to 2 mg/mL) to add to the semen samples. The optimal doses of TrKA and p75NTR antibodies were determined to be 32 and 1.5 μg/mL, respectively. The following treatments have been tested in in vitro and in in vivo semen traits:

- Control (diluted semen, C).
- NGF (100 ng/mL).
- NGF + aTrKA (100 ng/mL + 32 μg/mL, respectively).
- NGF + ap75NTR (100 ng/mL + 1.5 μg/mL, respectively).

### Quantification of NGF in seminal plasma

NGF concentration in seminal plasma was measured by ELISA, according to the manufacturer’s instructions (DuoSetELISA – R&D System, Milan, Italy) [25].

### Immunolocalisation of TrKA and p75NTR receptors

Ejaculated sperm were spread onto microscope slides, air dried at room temperature, fixed in absolute methanol for 10 min at − 20 °C. At RT, the slides were first permeabilised with 0.1% Triton–PBS for 20 min and then blocked with normal goat serum or normal horse serum (5%) in 0.1% Triton–PBS for 60 min. The slides were incubated with anti-TrKA (10 μg/mL AF175, R&D System) or anti-p75NTR (1 μg/mL MA5–13314, Thermo Fisher) at 4 °C, overnight. The cells were then washed three times for 10 min in PBS and incubated for 1 h at RT with the secondary antibody (5 μg/mL for TrKA: A-11034 Alexa Fluor 488 conjugated; for p75NTR: A-32723, Alexa Fluor 488 conjugated; Thermo Fisher). Negative controls were incubated with non-immune IgG diluted in PBS/BSA. After rinsing, samples were cover slipped with ProLong® Gold antifade reagent (Molecular Probes, IL, USA). TrKA- and p75NTR-positive cells were determined by using an epifluorescence microscope (BX-41, Olympus) equipped with a digital camera (F-viewer, Olympus) and Cell F imaging software (Olympus).

### FACSscan analysis of TrKA and p75NTR receptors

Aliquots of sperm were washed three times in PBS supplemented with 0.5% BSA (PBS/BSA) and centrifuged at 400 x g for 5 min. Subsequently, aliquots of 1 × 10^6^/mL of sperm were placed in FACSscan tubes and pre-incubated with PBS/BSA for 30 min at 4 °C, to minimise unspecific staining. Cells were then centrifuged, incubated for 1 h in PBS/BSA containing 2.5 μg/10^6^ cells of anti-TrKA (AF175, R&D System) and 2 μg/10^6^ cells of anti-p75NTR (MA5–13314, Thermo Fisher Scientific), at 4 °C. Afterwards, the cells were washed in PBS/BSA and incubated with the secondary antibodies (0,2 μg/mL ab72465 PE conjugated for TrKA and 2 μg/mL ab6785 FITC conjugated for p75NTR, Abcam, Cambridge, UK) for 30 min at 4 °C. After incubation, the cells were washed and rinsed in PBS/BSA. TrKA- and p75NTR-positive cells were quantified by FACSscan analysis. Ten thousand live-gated events were collected for each sample, and isotype-matched antibodies were used to determine binding specificity. The results were expressed as percentage of positive cells/antibody used for staining (% positive cells). All experiments included a negative control incubated with non-immune rabbit immunoglobulin IgG (1:10).

### Western blot analysis and immunoprecipitation of TrKA and p75NTR receptors

Aliquots of ejaculated sperm containing 8 × 10^7^ cells were washed once by centrifugation in PBS at 30,000 x g for 15 min at 4 °C, and the supernatants were discarded. Each pellet was suspended and then lysed with 1 mL of RIPA Lysis Buffer System, (Santa Cruz Biotechnology Inc.) for 20 min on ice. The mixture was then spun at 1000 x g (Eppendorf, USA) for 10 min, the supernatant was collected, and the total protein content was determined by the Bradford method following manufacturer’s instructions (Bio-Rad, CA, USA). Sample aliquots were stored at − 20 °C for western blot (WB) and immunoprecipitation analysis (IP).

For WB, samples were diluted with the sample buffer containing 50% glycerol, 20% sodium dodecyl sulphate (SDS), 0.5 M Tris–HCl (pH 6.8), 5% 2-mercaptoethanol, and 0.02% bromophenol blue, boiled for 5 min and loaded on 4–12% (w/v) SDS-PAGE gels [26]. Proteins were separated and transferred to nitrocellulose membranes using an iBlot™ 2 Dry Blotting System (Thermo Fisher) [27].

After blocking of the membrane with 5% dry milk in 10 mM Tris–HCl (pH 8), 0.5 M NaCl and 1% Tween-20 (TBS), membranes were incubated with primary antibodies overnight. After being washed with TBS containing 1% Tween-20, the blots were incubated with peroxidase conjugated secondary antibodies (HRP) and developed using electrochemiluminescence (ECL; Bio-Rad), according to the manufacturer’s instructions. In particular, the antibodies used were goat polyclonal anti-TrKA (1 μg/mL AF175, R&D System) and anti-goat secondary antibody (1:5000, Santa Cruz Biotechnology Inc.); mouse monoclonal anti-p75NTR (1.3 μg/mL MA5–13314, Thermo Fisher) and anti-mouse secondary antibody (1:5000, Santa Cruz Biotechnology Inc.); and rabbit polyclonal anti-βActin (1:500) and anti-rabbit secondary antibody (1:5000). Positive controls were HTB114 cells [28] and porcine Sertoli cells [29] for TrKA and p75NTR, respectively. Specific bands were detected by ECL. In order to confirm the specificity of the bands detected by WB, we performed a small-scale affinity purification of TrKA and p75NTR by IP, as previously described by Rossi et al. [30]. Briefly, we transferred 500 μg total cellular protein to a 1.5 mL microcentrifuge tube, added 5 μg of primary antibodies (AF175, R&D System) and mouse monoclonal anti-p75NTR (MA5–13314, Thermo Fisher) and incubated over night at 4 °C on a rocker platform. Then, we added 25 μL of Protein A/G PLUS-Agarose (Santa Cruz Biotechnology, Inc.) and incubated at 4 °C on a rocker platform for 3 h. We collected immunoprecipitates by centrifugation at 1000 x g for 5 min at 4 °C. The supernatants were carefully aspirated and discarded, the pellets were washed 4 times with 1.0 mL RIPA Lysis Buffer System (Santa Cruz Biotechnology Inc.), each time repeating the centrifugation step. After the final wash, we aspirated and discarded the supernatants and resuspended the pellets in 20 μL of electrophoresis sample buffer, followed by the run on a 4–12% (w/v) SDS-PAGE gel, transfer to a nitrocellulose membrane using an iBlot™ 2 Dry Blotting System (Thermo Fisher) as described above.

### Dose-effect of NGF on motility rate and track speed of sperm

The number of motile cells and the motion patterns of semen samples were analysed by computer-assisted sperm analyser (model ISAS, Valencia, Spain), with setup parameters already defined in previous experiments [31]. For each semen sample, two drops and six microscopic fields were recorded, for a minimum of 300 sperm tracks. The following sperm-motion parameters were reported: motility rate (%), the number of motile sperm divided by the sum of the motile plus immotile sperm within the field, and track speed (curvilinear velocity – VCL, μm/sec), the sum of the distances along the sampled path divided by the time taken by sperm to cover the track.

To determine the optimal dose of NGF (human NGF) on the in vitro motility rate and track speed of sperm, the sperm suspensions were treated with different doses of NGF (from 50 to 200 ng/mL) and analysed after 30 min of incubation under 5% CO_2_ at 37 °C. Three successive replications were conducted.

### Sperm capacitation patterns and acrosome reaction

The chlortetracycline (CTC) fluorescence assay was performed as reported by Cocchia et al. [32].

The CTC staining of the live sperm cells was examined under an epifluorescence microscope (OLYMPUS – CH_2_ excitation filter 335–425 and 480–560 nm for CTC and propidium iodide detection, respectively). Three distinct sperm fluorescence patterns were detected: fluorescence over the entire head, which is characteristic of intact cells (IC); a non-fluorescent band in the post-acrosomal region of the sperm head, which is characteristic of capacitated (CP) acrosome-intact cells; dull or absent fluorescence on the sperm head, which is characteristic of acrosome-reacted cells (AR). Three hundred sperm per sample were counted.

According to Castellini et al. [33], to evaluate the trend of IC, CP and AR, the CP/IC and AR/CP ratios were estimated. The first index (CP/IC) roughly estimates the pro/anti-capacitation effect, whereas the second index (AR/CP) measures the AR responsiveness.

### Determination of live, apoptotic and necrotic sperm

The detection of phosphatidylserine externalisation was performed by Annexin V Apoptosis Detection Kit (K101–100 BioVision CA, USA), made up of annexin V–fluorescein isothiocyanate (AnV–FITC) and propidium iodide–phycoerythrin (PI-PE), which are able to differentiate viable from necrotic and apoptotic cells.

The aliquots of experimental samples were washed with PBS, centrifuged, and suspended in 500 μL of Annexin-binding buffer to obtain a cell count of about 1 × 10^5^. Five μL of AnV–FITC and 5 μL of PI–PE (50 μg/mL) were added to each cell suspension.

The samples were incubated at RT for 5 min in the dark and then analysed by flow cytometer. Flow cytometry analysis was performed with a FACSscan Calibur (Becton Dickinson, CA, USA), by plotting green fluorescence (FL1)/AnV–FITC vs red fluorescence (FL2)/PI–PE positive cells. The combination of AnV and PI allows the discrimination of four sperm categories: viable cells (AnV−/PI-), early apoptotic cells (AnV+/PI-), late apoptotic cells (AnV+/PI+), and necrotic cells (AnV−/PI+). The sum of apoptotic cells was also calculated. Flow cytometry data acquisition was performed on a FACSscan Calibur equipped with 488 and 633 nm lasers and running CellQuest Software (Becton Dickinson, CA, USA). Ten thousand events were collected for each sample [34].

### Oxygen consumption

Oxidative phosphorylation and mitochondrial functionality were estimated by sperm oxygen consumption, following the method described by Castellini et al. [33]. Briefly, respiration (6 × 10^7^ cells) was evaluated in 0.5 mL of a solution of 120.6 mg/kg KCl, 2 mM K_2_HPO_4_, 0.025% BSA, 20 mM HEPES, at pH 7.4, and temperature equilibrated at 37 °C for 15 min prior to adding the substrates (5 mM succinate and 0.1 mM adenosine diphosphate - ADP). The rate of oxygen consumption was determined using a fibre optic oxygen monitor (Instech, USA) equipped with a probe fitted into a thermostatic water-jacketed chamber. Oxygen was sensed by fluorescence quenching of an indicator dye trapped in a matrix at the tip of the probe, as described by Macchioni et al. [35]. The oxygen content of the starting medium was normalised, assuming a concentration of about 190 nmol/mL at 37 °C.

### Statistical procedures

All the traits recorded were analysed with different linear models (StataCorp 14.0, 2015; Proc ANOVA). For the analysis of motility rate and VCL, a mixed linear model was used with NGF concentration (0, 25, 50, 75,100, 125, and 150 ng/mL) as fixed effect and buck as random effect.

The effect of neutralising receptors (aTrKA and ap75NTR) on the main physiological sperm traits (motility rate, VCL, capacitation, AR, apoptotic, necrotic and live cells) was evaluated with one-way linear model.

The significance of differences was evaluated by Bonferroni’s t-tests and differences were considered significant when *P* < 0.05. Least squares means (LS means) and standard errors (SE) are reported in tables and figures.

## Results

### Quantification of NGF in seminal plasma and analysis of TrKA and p75NTR receptors in ejaculated sperm

**Fig. 1:**
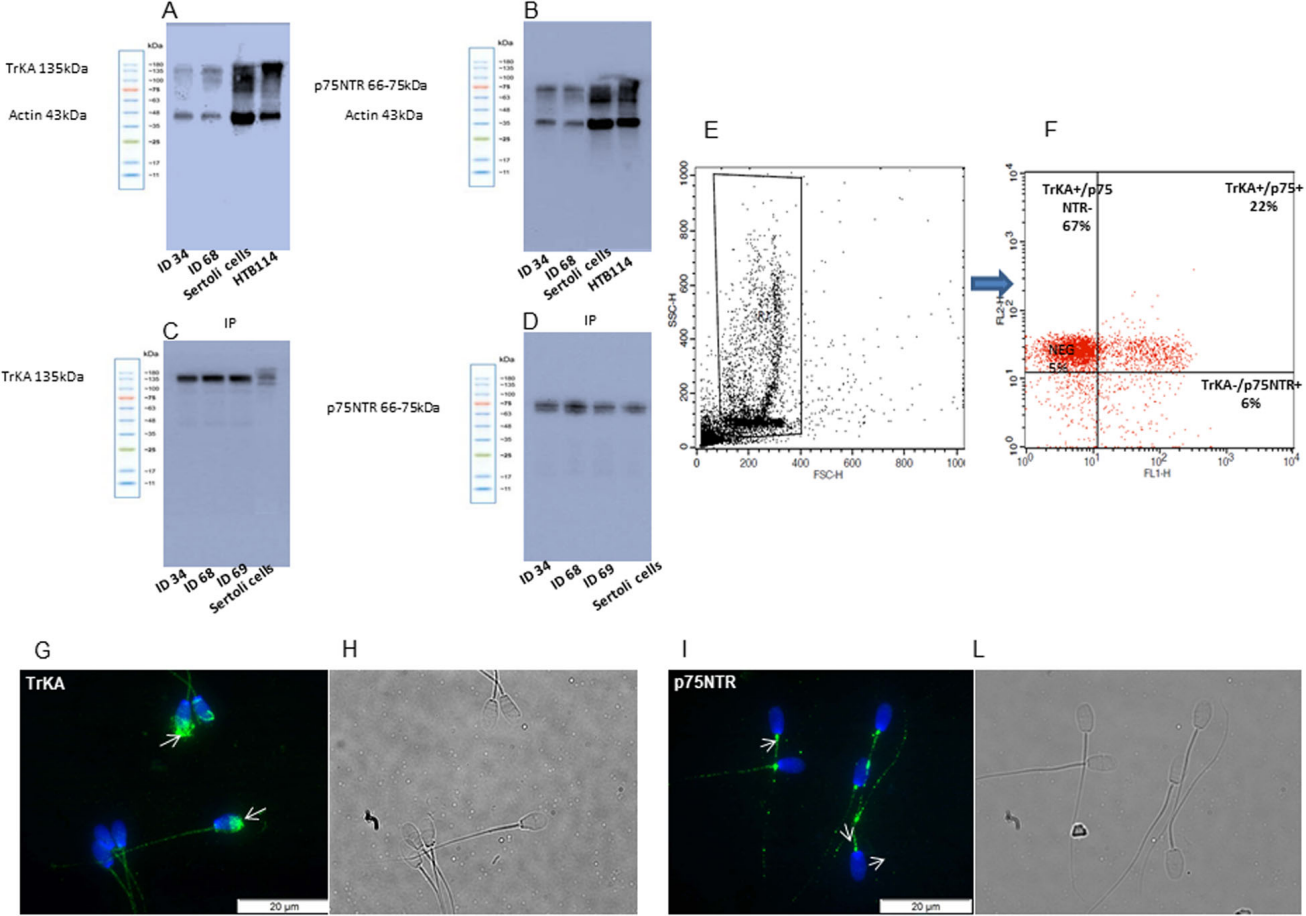
Protein expression and localization of TrKA and p75NTR receptors in ejaculated rabbit sperm. (**a**-**d**) Immunoblot of TrKA (**a**) and p75NTR (**b**). IP for TrKA (**c**) and p75NTR (**d**). ID 34, ID 68 and ID 69 are different sperm samples. (**e**) FSC/SSC dot plot obtained from a semen sample. A “flame-shaped region” (R1) is established to exclude debris, large cells and aggregates. (**f**) Right panel TrKA and p75NTR signals were recorded in the FL2-H and FL1-H channels, respectively. The upper left quadrant represents the TrKA-single positive cells, the upper right quadrant represents the TrKA/p75NTR-double positive cells, the lower left quadrant represents the double negative cells, and the lower right quadrant represents the p75NTR-single positive cells. (**g**-**l**) Immunolocalization of TrKA (**g**) and p75NTR (**i**) receptors in rabbit sperm. Fluorescent micrograph (**g**, **i**) and contrast phase (**h**, **l**) of sperm treated with anti-TrKA and anti-p75NTR antibody. Secondary antibodies are conjugated with Alexa Fluor 488 (green). Nuclei are counterstained with DAPI (blue). The images are representative of 3 separate experiments

NGF is widely present in the seminal plasma of rabbits (2288.75 ± 241 pg/mL). Both TrKA and p75NTR receptors were detected in the ejaculated rabbit sperm by several approaches (Fig .1). First, WB analysis showed a main band at 135 kDa for TrKA and a band ranging from 66 to 75 kDa for p75NTR (Fig. 1a and b). These data were successively confirmed by IP analysis (Fig. 1c and d) that showed well-defined bands similar to the control samples, endorsing the presence of these receptors in the sperm samples of rabbit.

Second, FACSscan analysis showed a high percentage of TrKA positive cells (92.6%) and a low percentage of p75NTR positive cells (26.5%) in raw ejaculated sperm (Fig. 1e and f).

Furthermore, immunofluorescence analysis confirms the presence of TrKA and p75NTR receptors in ejaculated spermatozoa. TrKA was not homogeneously distributed in all the samples and it was mainly found in the head (acrosome), whereas p75NTR staining was mainly present in the midpiece and tail (Fig. 1g and l).

The localisation of TrKA in the acrosome was also confirmed by FACSscan analysis. After induction with calcium ionophore, the TrKA positive cells decreased by about half (44.2%), whereas the p75NTR remained almost the same (Table 1).

**Table 1:** TrKA and p75NTR receptors in ejaculated sperm (raw and reacted-AR sperm) by FACSscan analysis

	% positive cells	
*Receptors*	TrKA	p75NTR
Raw sperm	92.6 ± 1.3	26.5 ± 4.4
AR	44.2 ± 0.8	25.5 ± 4.4

### Dose-effect of NGF on ejaculated sperm

**Fig. 2:**
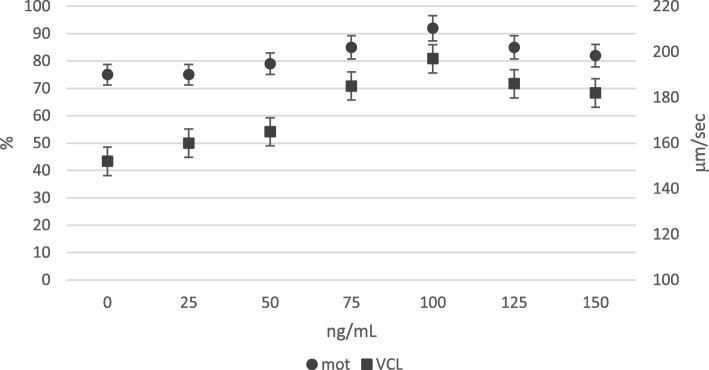
In vitro effect of NGF (ng/mL) dose on motility rate (%) and track speed VCL (μm/sec) of rabbit sperm (LSmeans ± SE; *n* = 12). Circle indicators mean motility rate (%); square indicators mean VCL (μm/sec)

One hundred ng/mL NGF significantly improved motility rate and VCL, with respect to lower doses; higher doses (125 and 150 ng/mL) did not further improve these sperm traits. Accordingly, the optimal dose used in the following experiments was 100 ng/mL (Fig. 2).

### Neutralisation of NGF receptors (TrKA and p75NTR) and semen traits

The neutralisation of TrKA receptors (NGF + aTrKA) did not modify sperm VCL and oxygen consumption, whereas it significantly reduced the motility rate (Table 2). On the other hand, the addition of ap75NTR (NGF + ap75NTR) reduced VCL without affecting the motility rate of sperm.

**Table 2:** Effect of in vitro treatment on percentage of motile sperm, track speed (VCL) and oxygen consumption

	Motility rate %	VCL μm/sec	Δ nmoli O_2_/min
C	76.0 b	232.5 b	5.96 a
NGF	88.2 c	255.2 c	6.26 b
NGF + aTrKA	60.2 a	233.2 b	6.58 b
NGF + ap75NTR	72.2 b	215.4 a	5.98 a
Pooled SE	5.2	15.2	0.3

Samples were diluted with TALP/BSA (C) or with 100 ng/mL NGF (NGF); 100 ng/mL NGF + 32 μg/mL aTrKA (NGF + aTrKA); 100 ng/mL NGF + 1.5 μg/mL ap75NTR (NGF + ap75NTR). Different letters in the same column(a..c) means: *P* < 0.05

Table 3 shows the effect of in vitro NGF treatment on sperm capacitation and acrosome reaction. With respect to the control, NGF increased capacitation and AR and the neutralisation of TrKA and p75NTR modified both traits. In particular, the addition of anti-p75NTR removed the stimulating effect of NGF on capacitation, whereas aTrKA resulted in nearly the same value of capacitation but significantly lower AR. Accordingly, the neutralisation of TrKA (NGF + aTrKA) inhibited the transition of capacitated sperm (~ 14%) to acrosome reaction (AR/CP = 17.2 vs 35.2 for the control), whereas ap75NTR reduced the progression of intact sperm towards capacitation (CP/IC = 13.7 vs 17.6 for the control).

**Table 3:** Effect of in vivo treatment on percentage of capacitated (CP), acrosome reacted (AR) and intact sperm (IC); apoptotic, necrotic and live sperm

	CP %	AR %	IC %	CP/IC	AR/CP	Apoptosis %	Necrosis %	Live cells %
C	14.2 b	5.0 bc	80.8b	17.6b	35.2b	7.8 ab	5.0 b	87.2 a
NGF	16.3 c	6.3 c	77.4a	21.1c	38.6b	7.0 a	3.8 a	91.0 b
NGF + aTrKA	14.5 b	2.5 a	83.0b	17.5b	17.2a	9.5 b	5.5 b	85.0 a
NGF + ap75NTR	11.4 a	4.3 b	84.3b	13.7a	37.4b	6.0 a	4.4 a	89.7 ab
Pooled SE	1.0	0.4	5.3	1.1	2.3	0.3	0.3	6.4

Samples were analysed after 2 h from dilution with TALP/BSA (C) or with 100 ng/mL NGF (NGF); 100 ng/mL NGF + 32 μg/mL aTrKA (NGF + aTrKA); 100 ng/mL NGF + 1.5 μg/mL ap75NTR (NGF + ap75NTR)

Different letters in the same column(a..c) means: *P* < 0.05

Regarding the survival profile (live cells, apoptosis and necrosis, Table 3) of sperm, NGF significantly increased the number of living cells and reduced necrosis, which appears mainly modulated by TrKA. Indeed, the neutralisation of TrKA (NGF plus aTrKA) increased apoptosis and necrosis, whereas apoptosis and necrosis lowered when p75NTR was blocked.

## Discussion

The common embryonic origin between brain and testis is an explanation for the presence of neural receptors in sperm. For instance, the process of acrosome reaction, a fundamental sperm function, includes several steps that recall the process of presynaptic secretion in neural cells [36].

Several authors have assessed the role of NGF and its receptors in the spermatogenesis of several animal species and humans [5, 6, 8, 15, 37]. Furthermore, recent papers have shown that NGF is involved in modulating the physiology of mature sperm, for example, the acrosome reaction and motility [18, 38–40]. However, most of the mechanisms and the role of NGF in sperm functions remain unclear.

The present study showed that rabbit seminal plasma contains a large amount of NGF (2288 ± 241 pg/mL) in comparison to humans (820 pg/mL) [15]. NGF amount in seminal plasma of rabbits was similar to previous reports [8, 41, 42]. It is possible that this high level of NGF in rabbit seminal plasma could be related to the mechanism of ovulation interacting with the sensory stimulation exerted by coitus, which is considered the main activator of luteinising hormone release [43]. Aside from the possible role of seminal NGF in the ovulation of rabbit does, our results confirm that NGF deeply affected most of the sperm cell functions.

Previous studies have identified the TrKA receptor in epididymal sperm of the golden hamster and man [15, 18] but, this appears to be the first time that p75NTR has been detected in ejaculated sperm of mammals. Our results indicate the presence of TrKA and p75NTR receptors in ejaculated sperm: p75NTR is mainly in the midpiece and tail, whereas TrKA resides in the head and acrosome. Li et al. [39] confirmed that TrKA was mainly localised in the head region and middle piece of bull spermatozoa. Sari et al. [44] found that TrKA receptors in sperm of the llama are localised in the middle piece and suggested that the localisation is species-specific. This discrepancy could also be explained by the fact that the permeabilisation of membrane with Triton, used before immunofluorescence, can affect the distribution/recognition of TrKA receptors [45].

The localisation of NGF receptors in sperm contributes somewhat to explaining their role. Indeed, through the detection and the neutralisation of NGF receptors, we have better defined the role of NGF on some crucial sperm functions: the kinetics, as prerequisite for egg fertilisation [46], the acrosome integrity [47, 48] and the viability [47].

The addition of NGF contributes to maintaining a high motility rate and track speed of sperm, whereas the blocking of both receptors affected the kinetic traits differently. Some researchers showed that NGF stimulated the motility rate of sperm [18, 39] but also the vigour of movement [40], whereas other authors, using frozen/thawed sperm, did not find a significant effect of NGF on mitochondrial activity [39]. Recently, Sanchez-Rodriguez et al. [43] confirmed that the addition of recombinant rabbit NGF significantly improved motility rate and VCL after 2 h of in vitro storage.

Other Growth Factors (GF) and their receptors have been characterized in somatic cells but there is scarce knowledge of their role in sperm. However, GF seem to have a significant role in sperm physiology: Saucedo et al. [49] showed that receptors of fibroblast GF were widely present in the acrosome and flagellum of sperm and exposure to these GF increased the phosphorylation of receptors and the activation of numerous kinases. Consequently, the incubation with these GF increases sperm motility, as well as sperm speed.

Recently, various other receptors (e.g. aromatase, androgen receptor, α-β estrogen receptors) have been identified in sperm of mammals. These receptors have been detected in human and ram sperm suggesting that the localization of receptors has a direct involvement in sperm capacitation, acrosome reaction and motility [50].

Moreover, the quantity and the distribution of receptors appeared linked to some male infertility disorders. Li et al. (2010) [15] showed that the seminal level of NGF in oligo- asthenozoospermic men is lower than in fertile men. The same for varicocele, which reduces the expression of α and b estrogen receptors and cancels the stimulus exerted by estradiol on capacitation and acrosome reaction [51].

The reduction of sperm track speed, attained by blocking p75NTR, was consistent with the high number of receptors found in the midpiece, which is the site of energy production by mitochondria. Thus, the reduction of sperm speed seems to be caused by lower energy production, confirmed by the lower oxygen consumption, through the mitochondrial respiratory chain [46].

However, the blocking of the p75NTR receptor did not affect the number of motile sperm; conversely, the blocking of the TrKA receptor affected the number of motile cells and did not modify sperm speed. This effect is partly correlated with the positive impact of NGF–TrKA on the survival rate of sperm, which decreased when TrKA was blocked.

NGF also affected the capacitation and AR of sperm; in turn, p75NTR neutralisation removed the stimulating effect of NGF on capacitation, whereas aTrKA reduced the acrosome reaction. Once more, this paper suggests that the process of capacitation and AR seems modulated by NGF through its receptors. Some authors [16] have found a positive effect of NGF on the AR without a distinction between capacitation and AR of hamster epididymal sperm (via TrKA) while other authors [39], using frozen/thawed semen of bulls, found no significant effect. Binding of NGF to the TrKA receptor results in kinase activation, such as the mitogen-activated protein kinase (MAPK) family and in particular of Ras/extracellular-signal-regulated protein kinase (ERK), which are AR modulators [39].

The response of sperm to NGF on sperm kinetics, capacitation and AR could be also related to the induction of apoptosis. The role of apoptosis in sperm cells is controversial and different from that observed in somatic cells [52]. Some authors [53] assessed that apoptosis is a process for deleting defective germ cells, mainly during spermatogenesis, while others [52] proposed supplementary roles as factors in regulating the lifespan of mature sperm.

Sperm apoptosis starts with the activation of mitochondrial enzymes that release different endonucleases going to the nucleus can induce DNA cleavage. Accordingly, apoptosis and DNA damage are mainly due to the Reactive Oxygen Substances (ROS) production of sperm [54], generated by the respiratory chain of the sperm [55, 56]. At the same time, capacitation [47], which allows the sperm to generate the propulsive force necessary for fertilisation [48], also appears to be ROS-dependent. Thus, the capacitation pathway could be regarded as similar to a pre-apoptotic status of sperm cells [57].

**Fig. 3:**
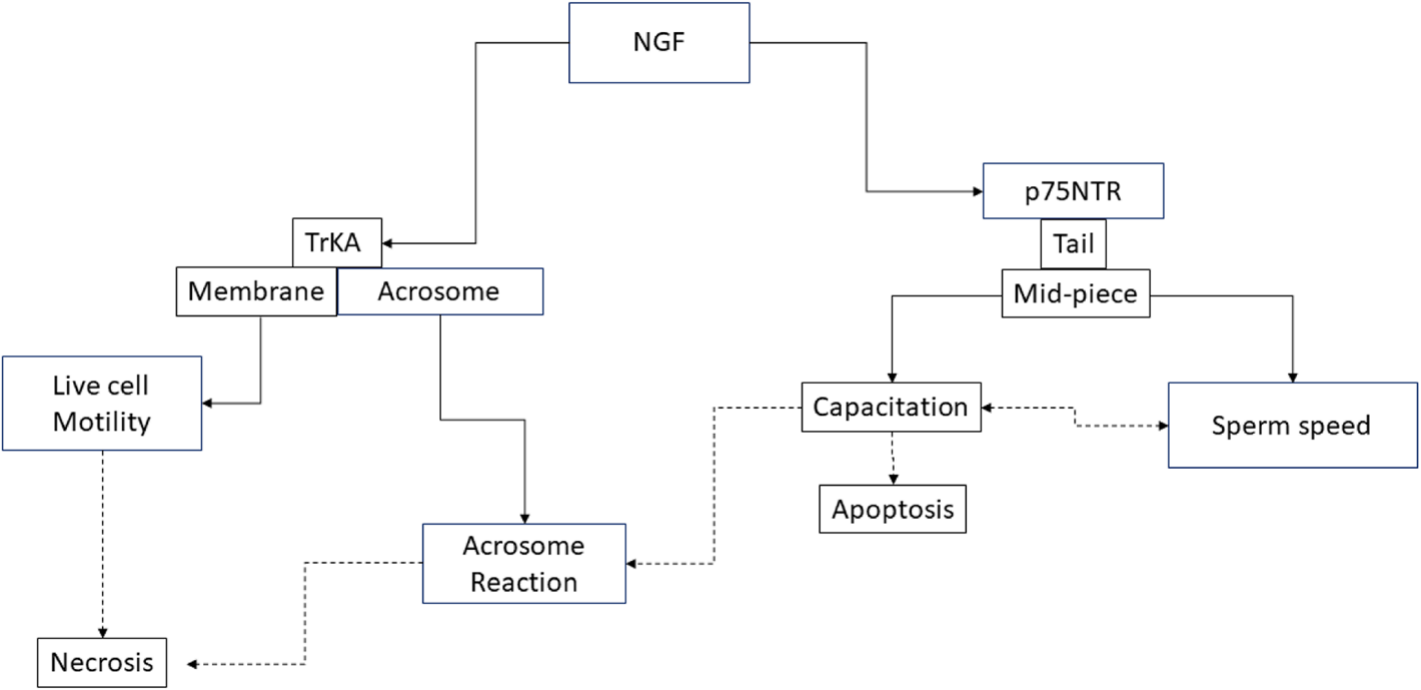
Possible role of NGF on the main semen traits trough the two receptors ways (TrKA and p75). Solid line means direct effects; dotted lines mean indirect effects

In this composite phenomenon, our results suggest that NGF, triggering mitochondria activity and the associated ROS production [58], contributes to modulate capacitation and sperm apoptosis mainly via p75NTR (Fig. 3).

Other authors [59] have reported that exogenous NGF influences viability, motility, nitric oxide release, and DNA fragmentation of sperm cells. NGF also modulates pro-survival factors, which normally prevent these cells from entering this pathway. The key to this process is the activation of the phosphatidylinositide 3–kinase (PI3–kinase) pathway, which maintains cell viability. It is reported that NGF, in other cell types, prevents chemically induced apoptosis through the activation of the PI3–kinase [28, 60].

Sperm cells used in artificial insemination are suspended in artificial media, but the cells lose their motility in 12–24 h [61]. The normal sperm survival in the female reproductive tract is much longer, as these cells must be able to survive for days. Evidently, other factors that are missing from our in vitro culture media are operating, and NGF could be one of them [62].

When NGF binds to the TrKA receptor, it drives the homodimerisation of the receptor, which causes the phosphorylation of the tyrosine kinase leading to the activation of PI3–kinase. Alternatively, the p75NTR receptor can form a heterodimer with TrKA, increasing the affinity and specificity for NGF [63]. Thus, NGF in sperm contributes to modulate survival, apoptosis and necrosis, depending on the receptors involved (TrKA – pro-survival and p75NTR – pro kinetic, apoptotic and necrosis), and could be considered a central factor regulating the senescence and the survival of sperm.

## Conclusions

In vitro supplementation of NGF to rabbit sperm improves some functional traits of cells. NGF affects kinetic and other physiological traits (capacitation, AR, apoptosis and necrosis) of sperm, and most of these changes are modulated by the receptors involved (TrKA or p75NTR). In particular, this is the first time that p75NTR has been detected in ejaculated sperm of mammals.

These findings provide new insights also on the human fertility viewpoint, considering that some seminal disorders have been correlated with a low NGF concentration in semen, and no studies have been done on the possible involvement of NGF receptors on seminal traits.

Other research is needed to deepen the knowledge of NGF and their receptors on physiological traits during sperm ageing.

## Data Availability

The datasets used and/or analysed during the current study are available from the corresponding author on reasonable request.

## Abbreviations

ALH: Amplitude of lateral head displacement
AnV: Annexin V
ap75NTR: p75NTR antibody
AR: Acrosome-reacted cells
aTrKA: TrKA antibody
BCF: Beat cross frequency
BSA: Bovine Serum Albumin
CaCl2: Calcium chloride
CP: Capacitated cells
CTC: Chlortetracycline
FITC: Fluorescein isothiocyanate
HEPES: 4-(2-hydroxyethyl)-1-piperazineethanesulfonic acid
HRP: Conjugated secondary antibodies
IC: Intact cells
IP: Immunoprecipitation analysis
K2HPO4: Dipotassium hydrogen phosphate trihydrate
KCl: Potassium chloride
LIN: Linearity
MgCl2: Magnesium chloride
Na2HPO4: Disodium hydrogen phosphate
NaCl: Sodium chloride
NaHCO3: Sodium bicarbonate
NGF: Nerve Growth Factor
p75NTR: p75 neurotrophin receptor
PBS: Phosphate buffered saline
PI-PE: Iodide-phycoerythrin
PKA: Protein kinase A
SDS-PAGE: Sodium Dodecyl Sulphate - PolyAcrylamide Gel Electrophoresi
TBS: Tween-20
TrKA: Tropomyosin receptor Kinase A
VCL: Curvilinear velocity
VSL: Straight line velocity
WB: Western blot
